# Therapeutic Activity of Lenalidomide in Mantle Cell Lymphoma and Indolent Non-Hodgkin's Lymphomas

**DOI:** 10.1155/2012/523842

**Published:** 2012-06-14

**Authors:** Marco Gunnellini, Lorenzo Falchi

**Affiliations:** S. C. Oncohematology, Perugia University, S. Maria, 05100 Terni, Italy

## Abstract

Mantle cell lymphoma (MCL) comprises 3–10% of NHL, with survival times ranging from 3 and 5 years. Indolent lymphomas represent approximately 30% of all NHLs with patient survival largely dependent on validated prognostic scores. High response rates are typically achieved in these patients with current first-line chemoimmunotherapy. However, most patients will eventually relapse and become chemorefractory with poor outcome. Alternative chemoimmunotherapy regimens are often used as salvage strategy and stem cell transplant remains an option for selected patients. However, novel approaches are urgently needed for patients no longer responding to conventional chemotherapy. Lenalidomide is an immunomodulatory drug with activity in multiple myeloma, myelodisplastic syndrome and chronic lymphoproliferative disorders. In phase II studies of indolent NHL and MCL lenalidomide has shown activity with encouraging response rates, both as a single agent and in combination with other drugs. Some of these responses may be durable. Optimal dose of lenalidomide has not been defined yet. The role of lenalidomide in the therapeutic armamentarium of patients with indolent NHL or MCL will be discussed in the present paper.

## 1. Introduction 

Non-Hodgkin's lymphomas (NHLs) are a heterogeneous group of lymphoid malignancies. The annual incidence of NHL in the United States is estimated to be 4.5% of all cancers, and they account for 3% of annual cancer-related deaths [1]. From a clinical and therapeutic standpoint, these neoplasias are subdivided into aggressive and indolent forms. Indolent lymphomas represent approximately 30% of all NHLs. Prognosis is correlated with the stage of the disease at the time of diagnosis, as well as to the international prognostic index (IPI) or other IPI-derived scores [2–5]. The current therapeutic approach for indolent NHL is based on the use of chemoimmunotherapy. Intensive treatments such as high-dose chemotherapy with autologous stem cell transplantation (ASCT) are typically reserved for relapsing patients whose disease is still chemosensitive [1]. 

Mantle cell lymphoma (MCL) comprises approximately 3 to 10% of NHL. It is a heterogeneous clinical entity with four recognized morphologic variants (i.e., classical, blastoid, pleomorphic and small cell, marginal zone-like). The small cell variant tends to be an indolent lymphoma, whereas both the blastoid and pleomorphic variants are associated with a clinical aggressive course. 

However, the majority (80%) of MCLs show intermediate characteristics. Thus, the median survival of the majority of patients is in the range of 3 to 5 years, and very few patients are cured [2]. 

MCL patients typically respond well to initial treatment with an overall response rate of approximately 90%. The addition of rituximab to conventional chemotherapy has even improved both quality and durability of responses either in newly diagnosed or relapsed disease [6, 7]. 

However, most patients will eventually relapse, with shorter and shorter disease-free intervals, and will require multiple different therapeutic interventions during the course of their disease [8, 9]. For this reason, there is a need for new effective agents with novel mechanisms of action to be tested in these patients.

## 2. Rationale for and Development of**** Lenalidomide in Lymphoproliferative**** Disorders 

Lenalidomide is an immunomodulatory drug (IMiD), derived from thalidomide, with increased potency and fewer side effects compared to its parent molecule. This agent has shown impressive clinical activity in patients with multiple myeloma (MM) [15] and has proven effective in chronic lymphocytic leukemia (CLL) [16] and T-cell lymphoma [17]. Preclinical models and preliminary clinical data also indicate significant antitumor activity of lenalidomide in B-cell malignancies [18, 19]. 

The mechanism of action of lenalidomide includes both immunomodulatory and nonimmunomodulatory effects [20–24]. It inhibits the production of proinflammatory cytokines (TNF-*α*, IL-1, IL-6, and IL-12) and enhances that of anti-inflammatory cytokine (IL-10) resulting in an increase of the tumor-cell apoptosis [20–22]. Lenalidomide also induces tyrosine phosphorylation of CD28, providing a costimulatory signal to T-cell activation by antigen-presenting cells via the B7 pathway [20–22]. IMiDs can decrease the expression of the angiogenic factors VEGF and IL-6 leading to a reduction of growth and survival of tumor cells [20–25]. Lenalidomide increases the number and Fc-*γ* receptor-mediated cytotoxicity of NK cells with an as-yet unclear mechanism of action [20]. Importantly, lenalidomide has also shown direct antiproliferative activity, in the absence of immune effectors, by decreasing erk1/2 and Akt2 and by inducing G0-G1 cell cycle arrest through inhibition of CDK2 activity [20–23]. Finally, in MM, lenalidomide has been shown *in vitro* to alter the microenvironment by downregulating cell surface adhesion molecules such as ICAM-1, VCAM-1, and E -selectin and inhibiting the adhesion of MM cell lines to the bone marrow stromal cells [20, 21]. 

## 3. Lenalidomide Monotherapy in**** Relapsed/Refractory Indolent and Mantle**** Cell Lymphoma 

Oral lenalidomide monotherapy produces durable responses in patients with NHL with a manageable toxicity profile (Table 1). In a pilot study of relapsed/refractory aggressive NHL, also including 15 and 5 stage III follicular lymphoma (FL) patients, lenalidomide induced an objective response rate of 35% with 12% complete responses/unconfirmed complete responses [10]. Patients enrolled in the study had received a median of 4 prior therapies. Fifty eight percent of patients were rituximab refractory. The most frequent G3 toxicity was neutropenia. A dose reduction was necessary in 18 (37%) patients (9 patients to 20 mg, 5 patients to 15 mg, 3 patients to 10 mg, and 1 patient to a 5 mg daily dose). Eight patients (16%) discontinued treatment because of adverse events. In a second trial [11] of heavily pretreated indolent NHL patients (median number of prior lines 3 (1–17)), single-agent lenalidomide resulted in an ORR of 23% (27% in follicular and 22% in small lymphocytic lymphoma). Median duration of response was not reached with a followup of 15 to 28 months. Median PFS for the whole group was 4.4 months (95% CI, 2.5–10.4). The most frequent G3 toxicities were hematological (neutropenia, thrombocytopenia, and anemia). Seventeen patients (40%) had a dose reduction (6 patients to 20 mg, 3 patients to 15 mg, 6 patients to 10 mg, and 2 patients to a 5 mg daily dose). Eight patients (19%) discontinued treatment because of adverse events, and 1 died on treatment due to sepsis. In another study of relapsed/refractory MCL, lenalidomide showed an overall response rate (ORR) of 43%. Twenty six percent of the patients had received stem cell transplantation and 32% had been exposed to bortezomib. ORR in these two groups was 53% and 57%, respectively. The most common grade 3 or 4 adverse event was neutropenia (43%) [12]. In a smaller study including 15 patients, 58% of whom being rituximab refractory, objective responses were achieved in 53% of cases with a 20% complete remission (CR) rate [13, 26]. Eight patients (53%) had a dose reduction, but only 1 patient discontinued treatment. Finally, Witzig et al. [14] treated 217 aggressive relapsed/refractory NHLs, including 26% of patients with MCL. Median number of prior chemotherapy lines was 3 (1–13). MCL patients showed an ORR of 42% with a median progression-free survival (PFS) not reached. Fifty three (53%) of patients required dose reduction (37 patients to 20 mg, 11 patients to 15 mg, 9 patients to 10 mg, and 10 patients to a 5 mg daily dose), and 23% discontinued lenalidomide. Most G3-G4 toxicities recorded were hematological (41% neutropenia, 19% thrombocytopenia, and 9.2% anemia). Ongoing trials of single-agent lenalidomide in refractory indolent NHL and MCL are summarized in Table 2. While in untreated patients a lenalidomide daily dose of 25 mg may be appropriate (see Table 1), in patients with relapsed or refractory disease, particularly if they are elderly and/or suffering from other comorbidities, a lower dose (15 to 20 mg) appears to be a wiser choice. Of note, because of the proliferation of phase II studies with different starting doses, and the dose-escalating design of several ongoing lenalidomide trials, a dose that is considered “reasonable” may not necessarily be the optimal one.

## 4. Lenalidomide in Combination for Relapsed/Refractory Indolent and MCL 

The Fc portion of rituximab mediates ADCC. Lenalidomide increases Fc-*γ* receptors on NK cell surface enhancing rituximab-mediated ADCC. Many trials have therefore evaluated the two drugs in combination (Tables 3 and 4). In a phase I/II study [27] of rituximab (375 mg/m^2^ weekly for 4 doses) and escalating doses of lenalidomide (from 10 to 25 mg daily on days 1–21 of 28-day cycles for a total of 6 cycles) in relapsed or refractory MCL, no responses were observed in the 10 mg and 15 mg groups, while patients receiving 20 mg daily achieved an ORR of 83%, including 17% of complete responses. At the dose of 25 mg, a G3 hypercalcemia and a lethal neutropenic fever were observed. The recommended lenalidomide daily dose to be used in combination with rituximab in phase II trials was therefore established to be 20 mg. Dutia et al. [28] tested rituximab (375 mg/m^2^ weekly for 4 doses plus 4 doses if no CR was reached) and lenalidomide (20 mg daily on days 1–21 of 28-day cycles) in heavily pretreated patients with indolent NHL. Treatment proved active and well tolerated (no dose reductions nor discontinuations were reported), particularly in patients with rituximab-refractory FL (response rate of 55%). In a recent phase II study that enrolled patients with MCL and either relapsed/refractory disease or ineligibility to intensive treatment, lenalidomide 25 mg daily for days 1–21 plus dexamethasone (40 mg on days 1, 8, 15, and 22) were given as postinduction consolidation therapy for 3 (patients in CR) or up to 12 (patients in partial remission/stable disease—PR/SD) cycles. Treatment was discontinued at CR or unacceptable toxicity. The study enrolled 33 patients. Median number of prior treatments was 3 (2–7). After a median followup of 16 months, median PFS and OS were 12 and 20 months, respectively, with median response duration of 18 months. Treatment was well tolerated, the most frequent toxicity being neutropenia (grade 3 in 25%, grade 4 in 28% of patients), leading to treatment interruption in two patients. Overall 9 serious adverse events were recorded, including one therapy-related fatal acute respiratory insufficiency [29]. Phase II trials are underway to test different combinations, notably including bortezomib plus lenalidomide [30].

## 5. Lenalidomide in Combination for Untreated Indolent and Mantle Cell Lymphoma 

The combination of rituximab and lenalidomide has also been tested in previously untreated patients with indolent NHL and MCL (Tables 5 and 6). In an ongoing study of 30 patients with advanced-stage indolent NHL and indication for treatment, [31] rituximab (375 mg/m^2^ on day 1 of each 28-day cycle) and lenalidomide (20 mg/day on days 1–21) for 6 cycles produced an ORR of 86% and an overall response rate (CRR) of 79%. Only 2 patients required treatment discontinuation due to toxicities leading the investigators to expand the originally planned accrual and include a total of 110 patients. In a study of 75 patients with indolent NHL [32], the same combination induced responses in 90% of patients with a 66% CRR. Only 5 patients discontinued treatment within the first two cycles due to toxicity. In a recent phase I trial [33], escalating doses of lenalidomide (from 5 to 25 mg once daily on days 1–14) were associated to R-CHOP21 (rituximab 375 mg/m^2^, cyclophosphamide 750 mg/m^2^, doxorubicin 50 mg/m^2^, vincristine 1.4 mg/m^2^ on day 1, and prednisone 100 mg/m^2^ days 1–5 every 21 days) for 6 cycles in untreated B-cell lymphomas. Lenalidomide 25 mg was established as the recommended dose. The most frequent toxicity was hematological (grade 3-4 neutropenia in 59% of patients), 6 patients experienced cycle delay, and 5 discontinued treatment, but no toxic death occurred. 

Strategies to build on the use of lenalidomide as a single agent appear the avenue to pursue. The role of dexamethasone is marginal, if any. On the contrary, the association of lenalidomide and rituximab appears to be feasible and shows encouraging activity in untreated and previously treated patients with indolent and MCL. The combination of lenalidomide with chemoimmunotherapy regimens such as R-CHOP is attracting, but both its feasibility and efficacy need to be tested in further prospective trials. Finally, the exploration of lenalidomide in other chemotherapy-free combination regimens is particularly fascinating and eagerly awaited. 

## 6. Lenalidomide as Maintenance Therapy for MCL 

With the aim of increasing disease control and survival, some authors have proposed a postinduction maintenance strategy for patients with MCL. One agent that proved successful in this context is rituximab [34]. Twenty-two untreated MCLs not candidate for autologous stem cell transplantation were treated with a maximum of 6 cycles repeated every 28 days of modified R-hyper-CVAD (rituximab, hyperfractionated cyclophosphamide, vincristine, doxorubicin, and dexamethasone) followed by rituximab maintenance (weekly doses every 6 months for a total of 4 courses). ORR and CRR were impressive (77% and 64%, resp.). In a recently presented multicenter phase III trial [35], 560 untreated elderly (>60 ys) patients not eligible for high-dose therapy were randomized to receive R-CHOP or rituximab, fludarabine, and cyclophosphamide followed by a maintenance phase with either rituximab or interferon-alfa. Rituximab maintenance doubled the remission duration compared to IFN (57% versus 26% at 4 years, resp., *P* = 0.0109). Not surprisingly, hematologic grade 3-4 toxicity was higher in the IFN arm. Overall survival did not differ between both maintenance arms (*P* = 0.17). Another randomized phase III trial evaluated the efficacy of lenalidomide versus placebo as maintenance therapy after first-line induction in patients with MCL not candidates for intensive treatment. Lenalidomide was given orally at the dose of 15 mg daily on days 1–21 every 28 days for 2 years, up to either disease progression or unacceptable toxicity, whichever occurred first. Only 9 patients (4 in CR and 5 in PR) were randomized (4 in the lenalidomide maintenance arm and 5 in the placebo). Two patients discontinued treatment due to toxicity and disease progression in the treatment and placebo arm, respectively. The study was prematurely terminated, and most analyses were not performed. A phase I/II Scandinavian trial [36] is ongoing in which lenalidomide is combined with rituximab (375 mg/m^2^ on day 1) and bendamustine (90 mg/m^2^ on days 1-2) as induction in untreated elderly (>65 years) MCL patients. Six 28-day induction cycles are followed by seven 28-day cycles of maintenance lenalidomide (25 mg daily on days 1–21). Recently, Ahmadi et al. [37] investigated the safety and efficacy of lenalidomide and rituximab in relapsed/refractory indolent or mantle cell lymphoma. Forty five sequential patients received two 28-day treatment cycles of lenalidomide 10 mg every day and four weekly doses of rituximab 375 mg/m2 in cycle 3 with (cohort 1) or without (cohort 2) weekly dexamethasone. In stable and responding patients, lenalidomide and dexamethasone (cohort 1) or lenalidomide alone (cohort 2) was continued until disease progression or unacceptable toxicity (median number of prior therapies was 3 (1–7)). Thirty five patients were evaluable for response. At a median followup of 11.8 months, PFS was 73% (95% CI: 53–86%), and ORR was 60% (12 CR; 9 PR). ORR did not differ between cohort 1 and cohort 2, **P* = 0.5*. Half of the patients temporarily suspended treatment, while 2 discontinued therapy. Several other trials of lenalidomide maintenance in patients with untreated or relapsed/refractory NHL are underway (Table 7).

## 7. Conclusions 

Lenalidomide is an immunomodulatory agent with remarkable activity in a variety of lymphoproliferative disorders. Its value is well established in multiple myeloma, and increasing evidence supports its role in the management of CLL patients. Phase II data are also delineating a role for lenalidomide in NHL, including MCL and indolent NHLs. Response rates are encouraging with response lasting 6.2 to 16.5 months in relapsed NHL when used alone. The drug is generally tolerated with hematological adverse events being the most common toxicity, and no unexpected toxicities in numerically limited trials the optimal dose of lenalidomide in maintenance or in combination with other agents remains to be defined. Single-agent lenalidomide 25 mg may be appropriate in young untreated patients, while a lower dose (15 to 20 mg) should be considered in relapsed/refractory elderly patients. The route is traced out, but informative, randomized phase III trials with careful study design and adequate patient numbers will be necessary to define the role of lenalidomide in the therapeutic armamentarium of patients with NHL.

## Figures and Tables

**Table 1:** Lenalidomide monotherapy trials.

Author	Year	Phase	Histology	*n*	Age (range)	Previous CT (range)	Rituximab refractory (%)	Schedule	ORR (%)	CR/CRu (%)	Median DR (months)
Wiernik et al. [10]	2008	II	Indolent NHL (10.2%)	49	65 (23–86)	4 (NR)	58	Lenalidomide 25 mg 21q28 days	60	20	6.2
			MCL (30.6%)						53	13.3	

Witzig et al. [11]	2009	II	Indolent NHL (100%)	43	63 (42–89)	3 (1–17)	67	Lenalidomide 25 mg 21q28 days	23	7	Not reached 16.5

Reeder et al. [12]	2009	II	MCL (100%)	54	69 (33–82)	3 (1–8)	NR	Lenalidomide 25 mg 21q28 days	43	17	NR

Habermann et al. [13]	2009	II	MCL (100%)	15	66 (45–84)	4 (2–7)	58	Lenalidomide 25 mg 21q28 days	53	20	Not reached 13.7

Witzig et al. [14]	2011	II	MCL (26.3%)	217	66 (21–87)	3 (1–13)	53.9	Lenalidomide 25 mg 21q28 days	42	21	Not reached 8.9

ORR: overall response-rate; CT: chemotherapy; CR/Cru: complete response/complete response unconfirmed; DR: duration of response; NR: not reported.

**Table 2:** Ongoing lenalidomide (L) monotherapy trials.

Name	Phase	Age	Histology	Drugs	Status
NCT00875667	II	>18 ys	Relapsed or refractory MCL	L	Ongoing and recruiting
NCT00737529	II	>18 ys	Relapsed or refractory MCL	L	Ongoing and recruiting
NCT00179673	II	>18 ys	Relapsed or refractory indolent NHL	L	Terminated

L: lenalidomide.

**Table 3:** Lenalidomide containing regimens in relapsed/refractory indolent and mantle cell lymphoma.

Author	Year	Phase	Histology	*n*	Age (range)	Previous CT	Combination	ORR (%)	CR (%)
Wang et al. [27]	2007	I/II	MCL	15	73 (62–84)	2 (1–7)	RL	83^∗^	17^∗^
Dutia et al. [28]	2009	II	Indolent NHL	15	60 (50–91)	4 (1–11)	RL	83.3	41
Zaja et al. [29]	2011	II	MCL	33	68 (51–80)	3 (2–7)	LD	52	24

^∗^: data from patients enrolled in lenalidomide 20 mg/daily arm. No response was obtained for lower dosage.

CT: chemotherapy; ORR: overall response rate; CR: complete response; RL: rituximab and lenalidomide; LD: lenalidomide and dexamethasone.

**Table 4:** Ongoing lenalidomide-based regime trials.

Name	Phase	Age	Histology	Drugs	Status
NCT01419795	II	>18 ys	Relapsed or refractory NHL after allo-SCT	RL	Ongoing and recruiting
NCT00238238	II	>18 ys	Relapsed follicular NHL	RL	Ongoing and recruiting
NCT00633594	I/II	>18 ys	Relapsed or refractory MCL	RVL	Ongoing and recruiting
NCT00553644 (27)	II	>18 ys	Relapsed or refractory MCL	LV	Ongoing and recruiting

RL: rituximab, lenalidomide; RVL: rituximab, bortezomib, lenalidomide; LV: lenalidomide, bortezomib.

**Table 5:** Lenalidomide containing regimens in untreated indolent and mantle cell lymphoma.

Author	Year	Phase	Histology	*n*	Age (range)	Combination	ORR (%)	CR (%)
Fowler et al. [31]	2010	II	Indolent NHL	30	56 (36–77)	RL	86	79
Samaniego et al. [32]	2011	II	Indolent NHL	75	57 (35–84)	RL	90	66
Tilly et al. [33]	2011	I	NHL	27	NR	RL-CHOP	96	74

ORR: overall response rate; CR: complete response; NR: not reported; RL: rituximab and lenalidomide; RL-CHOP: rituximab, lenalidomide, cyclophosphamide, doxorubicin, vincristine, prednisone.

**Table 6:** Ongoing lenalidomide-based regime trials.

Name	Phase	Age	Histology	Drugs	Status
NCT01415752	II	>60 ys	Untreated MCL	RBV + RL	Ongoing and recruiting
NCT01316523	II	>18 ys	Untreated indolent NHL	RL	Ongoing and recruiting
NCT00695786	II	>18 ys	Untreated indolent NHL	RL	Ongoing and recruiting
FIL R2-B	II	>18 ys	Untreated indolent NHL	RBL	Ongoing and recruiting

RBV: rituximab, bendamustine, bortezomib; RL: rituximab, lenalidomide; RBL: rituximab, bendamustine, lenalidomide.

**Table 7:** Ongoing lenalidomide-maintenance trials.

Name	Phase	Age	Histology	Drugs	Status
NCT01035463	I/II	>19 ys	Relapsed or refractory NHL	R-BEAM + ASCT + mL	Ongoing and recruiting
NCT01035463	I/II	>18 ys	Relapsed or refractory NHL	R-BEAM + ASCT + mL	Ongoing and recruiting
NCT01254578	I	>18 ys	High-risk hematologic cancers after Allo-SCT	mL	Ongoing and recruiting
NCT01045928	I/II	>18 ys	NHL	R + mL	Ongoing not recruiting
NCT01021423	III	>18 ys	Untreated MCL	FCR or R-CHOP + mL	Ongoing not recruiting
IIL MCL0208	III	18–60 ys	Untreated MCL	R-BEAM + ASCT + mL	Ongoing and recruiting

R-BEAM: rituximab, BCNU, etoposide, ara-C, melphalan; ASCT: autologous stem cell transplantation; mL: lenalidomide maintenance; R: rituximab; FCR: fludarabine, cyclophosphamide; rituximab; R-CHOP: rituximab, cyclophosphamide, doxorubicin, vincristine, prednisone.
